# Autotaxin-β interaction with the cell surface via syndecan-4 impacts on cancer cell proliferation and metastasis

**DOI:** 10.18632/oncotarget.26039

**Published:** 2018-09-04

**Authors:** Raphael Leblanc, Debashish Sahay, Audrey Houssin, Irma Machuca-Gayet, Olivier Peyruchaud

**Affiliations:** ^1^ Centre de Recherche en Cancérologie de Marseille, Marseille, France; ^2^ Institut Poli-Calmettes, Marseille, France; ^3^ INSERM, Unit 1068, Marseille, France; ^4^ University Aix-Marseille, Marseille, France; ^5^ Department of Medicine, Colombia University Medical Center, New York City, NY, USA; ^6^ INSERM, Unit 1033, Lyon, France; ^7^ Université Claude Bernard Lyon 1, Lyon, France

**Keywords:** autotaxin, heparan sulfate proteoglycans, syndecan-4, adhesion, metastasis

## Abstract

Autotaxin (ATX) promotes cancer cell metastasis through the production of lysophosphatidic acid (LPA). ATX binds to αvβ3 integrins controlling metastasis of breast cancer cells. We screened a series of cancer cell lines derived from diverse human and mouse solid tumors for the capacity of binding to ATX and found only a modest correlation with their level of αvβ3 integrin expression. These results strongly suggested the existence of another cell surface ATX-interacting factor. Indeed, ATXα has been shown to bind heparan-sulfate chains because of its unique polybasic insertion sequence, although the biological significance is unknown. We demonstrated here, that among all cell surface heparan-sulfate proteoglycans, syndecan-4 (SDC4) was essential for cancer cell interaction with ATXβ but was restrained by heparan-sulfate chains. In addition, exogenous ATXβ-induced MG63 osteosarcoma cell proliferation required physical interaction of ATXβ with the cell surface via an SDC4-dependent mechanism. In a preclininal mouse model, targeting SDC4 on 4T1 mouse breast cancer cells inhibited early bone metastasis formation. Furthermore, SDC4-prometastatic activity was totally abolished in absence of ATX expression. In conclusion our results determined that ATX and SDC4 are engaged in a reciprocal collaboration for cancer cell metastasis providing the rational for the development of novel anti-metastasis therapies.

## INTRODUCTION

Autotaxin (ATX) is a unique member of the nucleotide pyrophosphatases phosphodiesterase family because of its characteristic lysophospholipase D (lysoPLD) activity that is responsible for the synthesis of lysophosphatidic acid (LPA) from lysophosphatidylcholine (LPC) and other lysophospholipid precursors [1]. LPA activates a large series of six LPA receptors (LPA_1–6_) that contributes to cell proliferation, survival and/or cytoskeletal rearrangement and motility [2]. ATX was originally characterized as an autocrine motility factor produced by melanoma cells due to its lysoPLD activity, suggesting its contribution in cancer cell migration/invasion [3, 4]. Expression of ATX is elevated in multiple cancer types [4–12] and experimental forced expression of ATX or LPA receptors (LPA_1_, LPA_2_, LPA_3_) in the mammary gland of MMTV-driven gene transgenic mice induces the development of spontaneous breast tumors and metastasis formation, reinforcing the importance of the ATX/LPA track in oncogenesis and metastasis [13]. Intriguingly, in the blood circulation ATX (5 µg/mL), LPA (0.1 µM) and LPC (400 µM) are represented at substantially high levels [14, 15]. However, the pathophysiological significance of such high systemic levels of ATX, LPA and LPC is not well understood. The recent resolution of ATX crystal structure led to the identification of amino acids E^109^ and H^117^ located in the somatomedin B-like domain 2 that are involved in ATX binding to β3 integrins (αvβ3, αIIbβ3) [16, 17]. This binding has a functional impact on ATX as it increases lysoPLD activity [17], promotes cancer cell motility [18] and metastasis [19]. These studies supported the concept for a novel biological way of action of ATX involving the binding to β3 integrins that might localize ATX activity to the cell surface, providing a favorable environment for LPA production and action in the close vicinity of its cell surface receptors.

Five ATX isoforms (α,β,γ,δ,ε) have been identified so far [20]. However, their specific role in any pathophysiological processes has not been characterized yet. ATXα binds to heparin-sulfate (HS) chains of HS proteoglycans (HSPG) due to its unique insertion of a 52 polybasic amino acid sequence; however, the biological significance of this domain is unknown [21]. ATXβ is the isoform expressed by cancer cells that binds to αvβ3 integrins but ATXβ is also expressed by endothelial cells of high endothelial venules and binds to α4β1 integrins, impacting on T-lymphocyte migration into secondary lymphoid organs [22].

Due to the specialization of tissues and organs, most cells express different repertoires of adhesive molecules conferring specific responses upon external stimulations. Besides expression of LPA receptors, it is conceivable that expression of specific cell surface adhesive molecules may limit or restrict the action of ATX. In this study we ask to know whether other types of adhesive molecules than integrins and HS chains could contribute to ATX binding to cell surface. For this study we used ATXβ as a source of recombinant protein because it is the most abundant isoform in human and mouse [23]. We identified HS proteoglycan syndecan-4 (SDC4) as a new molecule that controls ATXβ interaction with cancer cell surface through a domain located in SDC4 core protein. Using a syngenic mouse model of breast cancer metastasis, we determined that SDC4 controls early steps of the metastasis process through an ATX-dependent mechanism.

## RESULTS

### Binding of ATXβ to cancer cells is partially mediated by αvβ3 integrin

**Figure 1:**
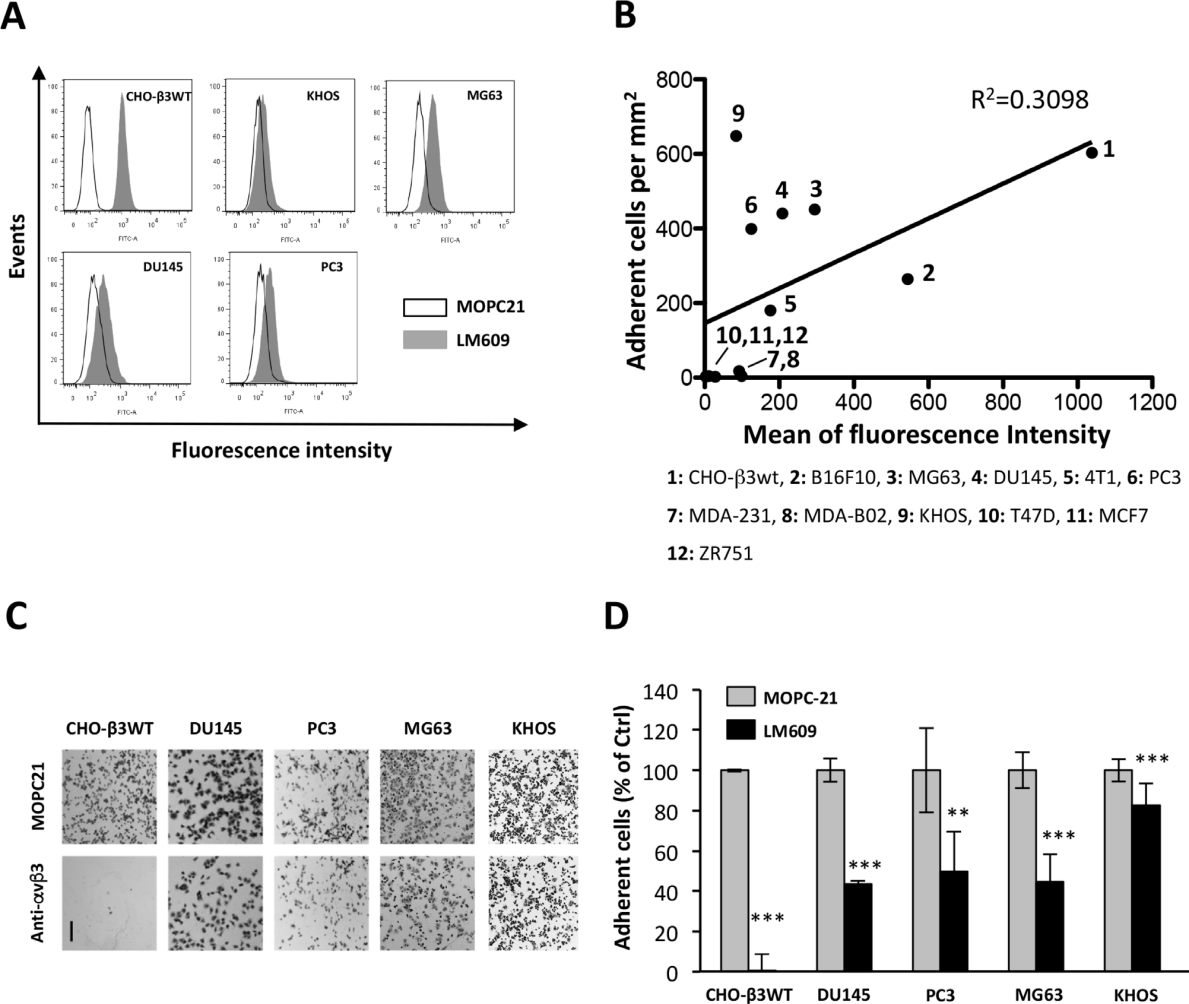
Integrin αvβ3 is partially involved in cell binding to ATXβ (**A**) Flow cytometry detection of cell surface expression of αvβ3 integrin in CHO-β3WT, KHOS, MG63, DU145 and PC3 cells. Cells were immunostained with LM609 monoclonal antibody (black bar) or isotype control antibody MOPC21 (open bar). (**B**) Linear regression analysis for the cell surface expression of αvβ3 integrin evaluated by flow cytometry (expressed in mean of fluorescence intensity) and the level of cell interaction with ATXβ evaluated by cell adhesion assay on ATXβ-coated plates (expressed in adherent cell number per mm^2^). Human, murine and ovarian cell lines are numbered from 1 to 12. (**C**–**D**) Inhibition of cell adhesion on ATXβ with LM609 antibody (anti-αvβ3). Indicated cell lines were preincubated for 1 h in the presence of LM609 or MOPC21 antibodies (10 µg/mL). (C) Representative images of cell adhesion plates for indicated cell lines. Scale bar represents 200 µM. (D) Data represent the mean ± SD of adherent cells (in % of MOPC21-treated cells) of 3 experiments performed in 8 replicates (^**^P < 0.01; ^***^P < 0.001, using 1-way ANOVA with a Bonferroni post-test).

ATX binds to β3 integrin family members (αIIbβ3, αvβ3) [17, 19]. Integrin αvβ3 is wildly expressed among different cell types and tissues including in cancer; whereas αIIbβ3 integrin is restricted to the megakaryocyte lineage [24]. To determine whether involvement of αvβ3 integrin in ATXβ binding was a commune feature among different types of cancers, we selected a series of human and murine cancer cell lines from melanoma, osteosarcoma, breast and prostate cancers. We first evaluated the level of expression of αvβ3 integrin in each cell line by flow cytometry using LM609 monoclonal antibody (Figure 1A). Then all cell lines were tested for their capacity of adherence to ATXβ-coated plates. The linear regression calculated from the level of αvβ3 integrin (mean of fluorescence) and the number of adherent cells per mm^2^ indicated that there was a positive trend but no strict correlation between these two parameters (*R*^2^ = 0.3098, *p* < 0.05) (Figure 1B). All human cell lines were then incubated with LM609 antibody before adhesion assays. In this condition, LM609 inhibited cell binding to ATXβ that reached a maximum of 60% of inhibition on human prostate DU145 cancer cells (Figure 1C–1D). CHO-β3WT cells were used as a positive control since they have been genetically manipulated allowing high expression of human αvβ3 integrins (Figure 1B) [25]. Intriguingly, human osteosarcoma KHOS cells exhibiting the lowest levels of αvβ3 integrins at their cell surface (Figure 1B) had the significantly highest capacity of adherence to ATXβ (Figure 1B). Also, LM609 treatment inhibited the binding of KHOS cells to ATXβ but to a much lower extent of 15% (Figure 1C–1D). These results support the existence of complementary mechanisms that in addition to αvβ3 integrins are involved in ATXβ binding with the cell surface.

### HS chains restrain cell interactions with ATXβ

**Figure 2:**
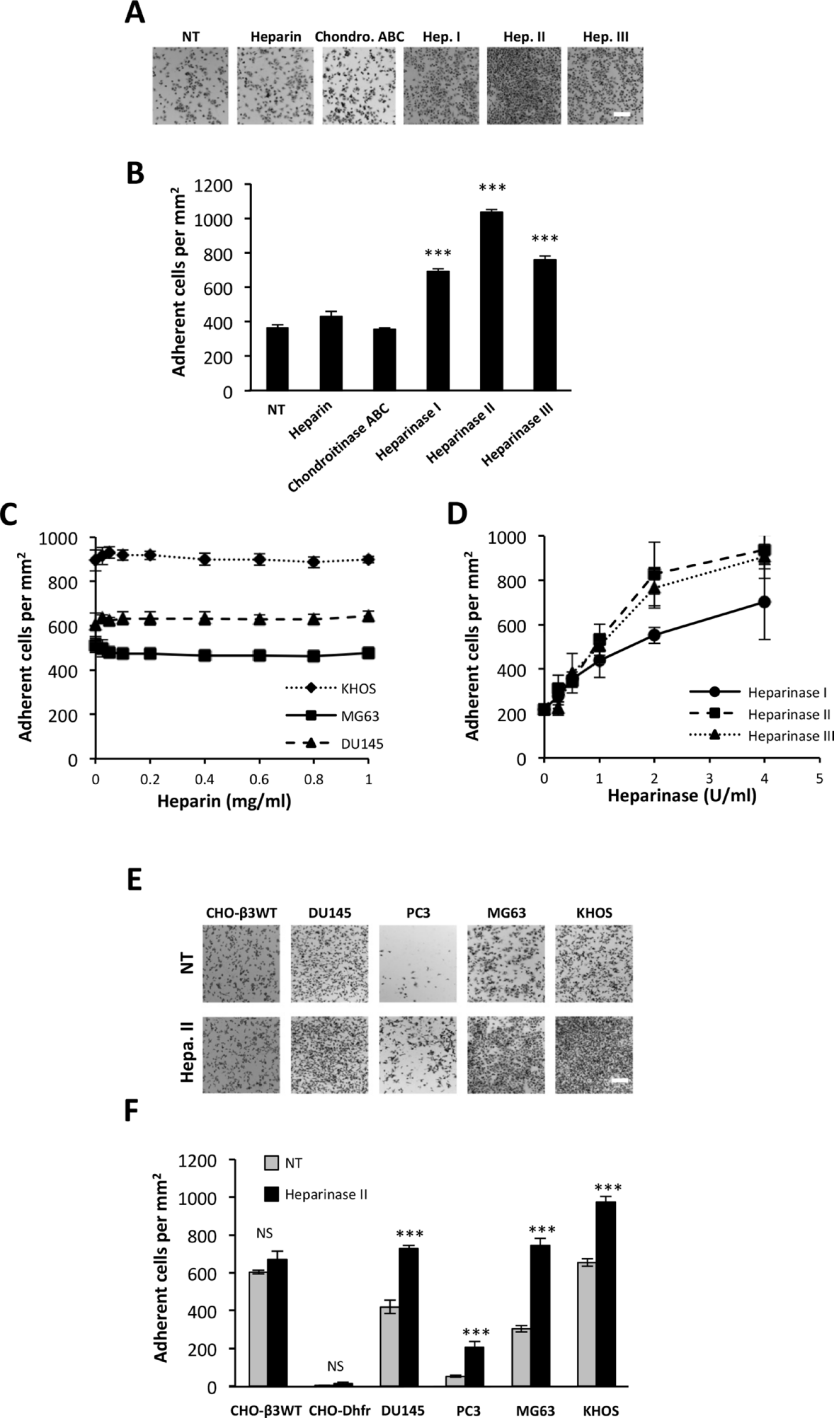
Heparan sulfate chains restrain cell interactions with ATXβ (**A**–**B**) Effect of treatment with heparin, chondroitinase ABC (Chondro.ABC), heparinase I (Hep.1), heparinase II (Hep.II) or heparinase III (Hep.III) on MG63 cell adhesion to ATXβ. NT: Nontreated cells (A) Representative images of MG63 cell adhesion. Scale bar represents 200 µM. (B) Data represent the mean of adherent cells/mm^2^ ±SD of adherent cells of at least 3 experiments performed in 8 replicates (^***^P < 0.001, using 1-way ANOVA with a Bonferroni post-test). (**C**) Effect of increased concentrations of heparin on MG63, KHOS and DU145 cell adhesion to ATXβ. Data represent the mean of adherent cells/mm^2^ ± SD of adherent cells of 3 experiments performed in 8 replicates. (**D**) Effect of increased concentrations of heparinase I, II or III on MG63 cells adhesion to ATXβ. Data represent the mean of adherent cells/mm^2^ ±SD of adherent cells of 3 experiments performed in 8 replicates. (**E**–**F**) Effect of Hep.II on CHO-β3WT, CHO-Dhfr, KHOS, MG63, DU145 and PC3 cell adhesion to ATXβ. (E) Representative images of cell adhesion. Scale bar represents 200 µM. (F) Data represent the mean of adherent cells/mm^2^ ±SD of adherent cells of at least 3 experiments performed in 8 replicates (^***^P < 0.001, NS: not significant, using 1-way ANOVA with a Bonferroni post-test).

Among potential partners, we tested the potential involvement of HS chains in the interaction of ATXβ to cancer cell surface. We carried out cell adhesion assays using human osteosarcoma MG63 cells on ATXβ-coated plates. Assays were run in presence of heparin or after cell pretreatment with chondroitinase ABC or heparinase I, II or III. Heparin and chondroitinase ABC had no effect on the number of MG63 cells bound to ATXβ, indicating that ATXβ did not bind to HS and chondroitin chains (Figure 2A–2B). Absence of effect of heparin was not due to a subeffective dose as judged by the absence of effect of increased concentrations of heparin on MG63 cell binding (Figure 2C). In addition, the absence of effect of heparin was not restricted to MG63 cells as this was also observed using human osteosarcoma KHOS and human prostate cancer DU145 cells (Figure 2C). Interestingly, treatment of MG63 cells with heparinases I, II or III increased by 2- to 2.5-fold the binding of MG63 cells on ATXβ (Figure 2A–2B). This effect was dose-dependent and saturable, suggesting the specificity of the phenomenon (Figure 2D). Furthermore, increased binding following heparinase II treatment was found on all cancer cell lines tested (Figure 2E–2F). These results indicated that HS interfered with ATXβ interaction with the cell surface but without a direct interaction with ATXβ.

### SDC4 mediates cell interaction to ATXβ

**Figure 3:**
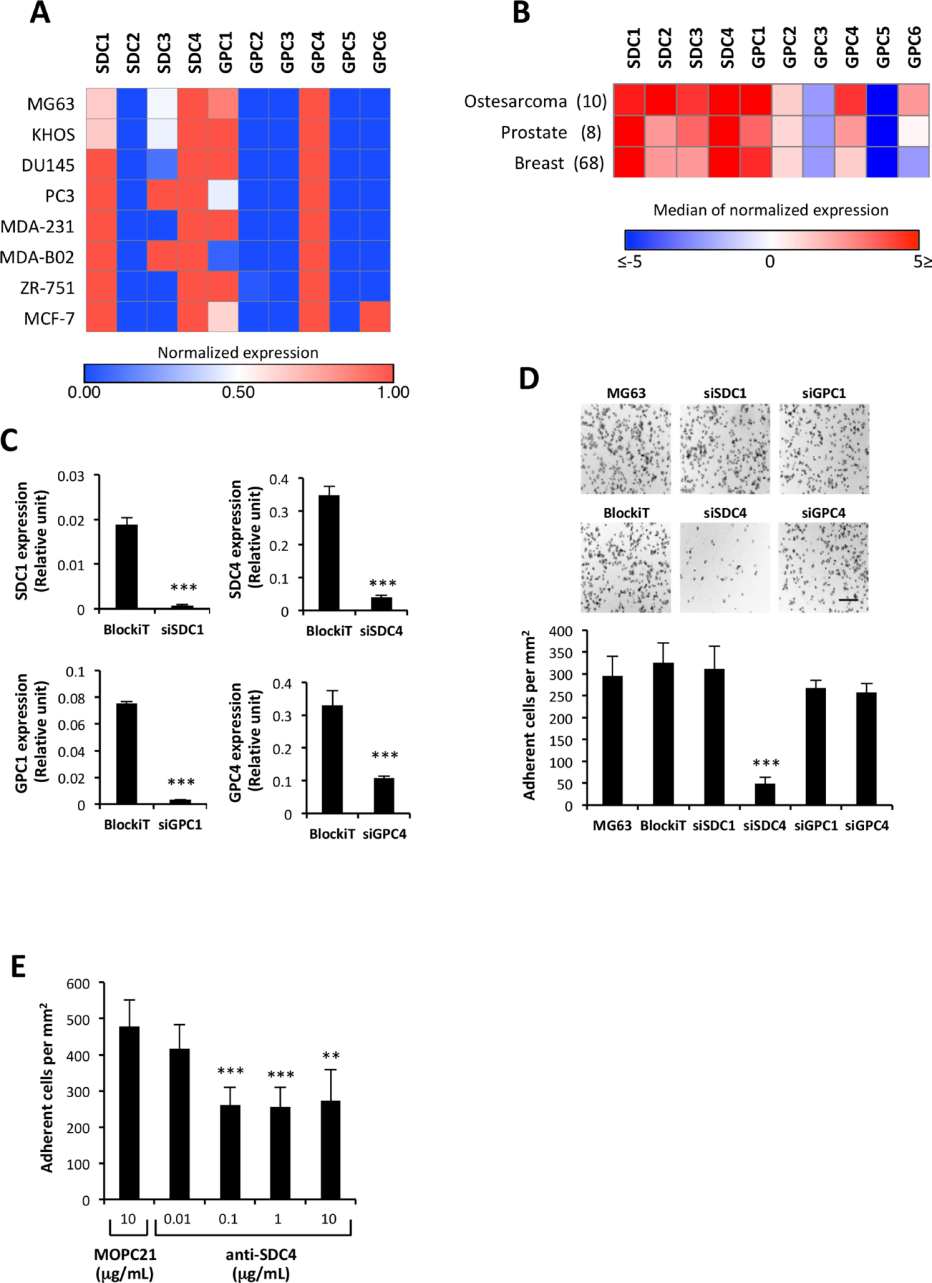
SDC4 mediates cell interaction to ATXβ (**A**) mRNA expression of cell surface heparan sulfate proteoglycan, syndecans (SDC) and glypicans (GPC) in human osteosarcoma (MG63, KHOS), prostate (DU145, PC3) and breast cancer (MDA-MB-231, MDA-B02, ZR751, MCF7) cell lines. For each gene, the expression has been normalized to L32 and represented on a heat map. High expression is in red and low expression in blue. (**B**) Expression of SDC and GPC genes in a large panel of cancer cell lines from osteosarcoma, prostate and breast cancers. Median values of normalized expression of each gene were extracted from the Cancer Cell Line Encyclopedia (CCLE [26]). Numbers indicate total numbers of cell lines in each category. High expression is in red and low expression in blue. (**C**) Real-time PCR analysis of SDC1, SDC4, GPC1 or GPC4 mRNA expression in MG63 cells transfected with sSDC1, siSDC4, siGPC1 or siGPC4. Values have been normalized to the housekeeping L32 gene. Data represent the mean ± SD of 2 independent experiments performed in triplicate (^***^P < 0.001, using Mann-Withney test).(**D**) Effect of silencing SDC1, SDC4, GPC1 or GPC4 on MG63 cell adhesion to ATXβ. (Upper panels) Representative images of cell adhesion culture plates. Scale bar represents 200 µM. **(**Lower panel) Data represent the mean of adherent cells/mm^2^ ±SD of adherent cells of at least 3 experiments performed in 8 replicates (^***^P < 0.001, using 1-way ANOVA with a Bonferroni post-test). (**E**) Inhibition of MG63 cell adhesion on ATXβ with anti-SDC4 antibody. Cells were preincubated for 1 h in the presence of anti-SDC4 (5G9) or MOPC21 antibodies. Data represent the mean of adherent cells/mm^2^ ±SD of 3 experiments performed in triplicate (^**^P < 0.01, ^***^P < 0.001 *vs*. cells treated with MOPC21 antibody using 1-way ANOVA with a Bonferroni posttest).

To determine if one specific type of cell surface HSPG (CS-HSPG) could be responsible for the negative action of HS on ATXβ cell binding, we first screened by real-time PCR the expression levels of mRNAs for all 10 CS-HSPG (4 SDC; 6 GPC) in human osteosarcoma, prostate and breast cancer cell lines. We found that SDC1, SDC4, GPC1 and GPC4 were the most communally highly expressed types of CS-HSPG among these cells (Figure 3A). In order to avoid misinterpretations due to the limited number of cell lines in each cancer category we extracted the median values of normalized expression of each gene from the Cancer Cell Line Encyclopedia (CCLE) [26] for each cancer entity compiling 10 cell lines from osteosarcoma, 8 from prostate cancers and 68 from breast cancers. This analysis confirmed our previous observation (Figure 3B). Then we used a siRNA strategy to silence the expression of each identified CS-HSPG using MG63 cells (Figure 3B). Transfected cells were then tested for their capacity of interaction with ATXβ in the cell-binding assay. Surprisingly, as opposed to what was expected, none of transfectants exhibited any enhanced binding capacity (Figure 3C–3D). Furthermore, specific silencing of SDC4 significantly inhibited MG63 cell interaction with ATXβ (Figure 3C–3D). Likewise, preincubation of MG63 cells with an anti-SDC4 blocking antibody also significantly decreased cell adhesion (Figure 3E). Even unexpected and somehow contradictory with previously determined action of HS chains on ATXβ cell binding, these results suggested that SDC4 core protein was essential for MG63 cell interaction with ATXβ, involving at least part of the SDC4 extracellular domain.

SDC4 mRNA is expressed at high levels in almost all primary tumor entities (Supplementary Figure 1A), as opposed to ENPP2 mRNA whom highest levels could be found in renal cancers and melanoma (Supplementary Figure 1B), two cancer types that were not addressed in our study. Unfortunately, osteosarcoma clinical data were not available from the TCGA database [27]. Complete Kaplan–Meier curves and Cox analyses on all cancer entities would be required to evaluate whether or not combined expression of both ENPP2 and SDC4 could predict overall- or disease-free patients survival. Our first evaluation on overall survival of patients with breast tumors showed a trend that did not reach statistically significance with a poor prognosis value of ENPP2^high^/SDC4^high^ versus ENPP2^low^/SDC4^low^ (Supplementary Figure 2).

**Figure 4:**
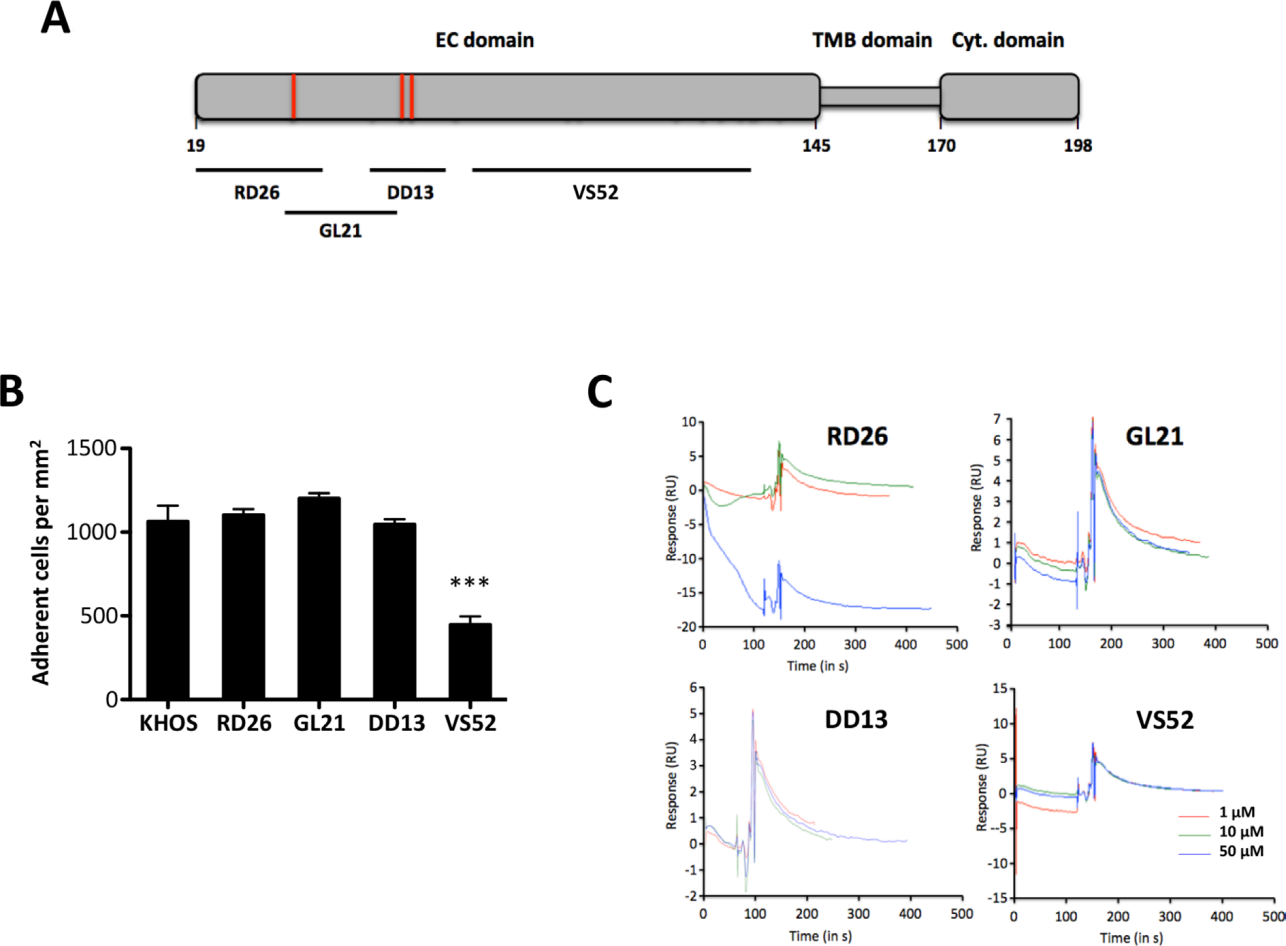
An extracellular domain SDC4-derived peptide controls cell adhesion on ATXβ but does not interact directly with ATXβ (**A**) Schematic representation of human SDC4 protein. Numbers referred to amino acid position in the primary sequence. Extracellular (EC) domain, Transmembrane (TMB) domain and Cytoplasmic (Cyt.) domain. Red bars indicate heparan-sulfate chain binding sites. Black bars indicate covering areas of defined peptides in SDC4 EC domain (RD26, GL21, DD13, VS52). (**B**) Competition assay of KHOS cell adhesion on ATXβ in presence of RD26, GL21, DD13 and VS52 SDC4-derived peptides. Cells were preincubated for 1 h in the presence of indicated peptides (50 µg/mL). Data represent the mean of adherent cells/mm^2^ ±SD of one experiment representative of 3 experiments performed in 8 replicates (^***^P < 0.001 *vs*. KHOS cells using 1-way ANOVA with a Bonferroni posttest). (**C**) Binding of SDC4-derived peptides to ATXβ studied by surface plasmon resonance. Panels represent normalized sensorgrams corresponding to specific binding of RD26, GL21, DD13 and VS52 peptides injected at indicated concentrations over 120 sec at a rate of 30 µL/min over both ATXβ-bound and the reference surfaces of the NTA sensor chip. Dissociation was measured for 200 sec.

We then explored whether SDC4 core protein interact directly or indirectly with ATXβ. We designed four synthetic peptides, RD26, GL21, DD13 and VS52 (Table 1) encompassing the whole 126 amino acid sequence of the SDC4 extracellular (EC) domain (Figure 4A). Synthetic peptides will not incorporate post-translational modifications such as linkage to HS chains, partially mimicking cell treatment with heparinases. We used KHOS cells because they have the highest binding capacity to ATXβ (see Figure 1B). Based on a competition assay we found that RD26, GL21 and DD13 peptides did not interfere with cell binding to ATXβ (Figure 4B). In contrast, VS52 peptide potently inhibited the binding by 50%. This result was surprising because this specific region of SDC4-EC domain was shown to control cell adhesion mediated through integrins but has not been involved so far in ligand interaction [28, 29]. To further characterize potentially direct interaction between SDC4 and ATXβ we carried out Surface Plasmon Resonance using ATXβ immobilized on a NTA sensor chip and individual peptides as analytes. As shown on Figure 4C none of peptides, tested either at 1 µM, 10 µM or 50 µM bound significantly to ATXβ (Figure 4C). Altogether, these results strongly suggested that SDC4 and ATXβ did not interact directly. Nevertheless, VS52 sequence of SDC4-EC domain appeared as a functionally active domain in ATXβ cell binding mediated through an uncharacterized partner.

**Table 1:** List of SDC4-derived peptides

Peptide name	Location^(a)^	Size
RD26	22–47	26 aa
GL21	40–60	21 aa
DD13	56–60	13 aa
VS52	79–130	52 aa

(a) Amino acid location was defined from human SDC4 sequence (https://www.uniprot.org, Entry: P31431). aa: amino acid.

### Blocking SDC4 impairs ATXβ-induced cell proliferation

**Figure 5:**
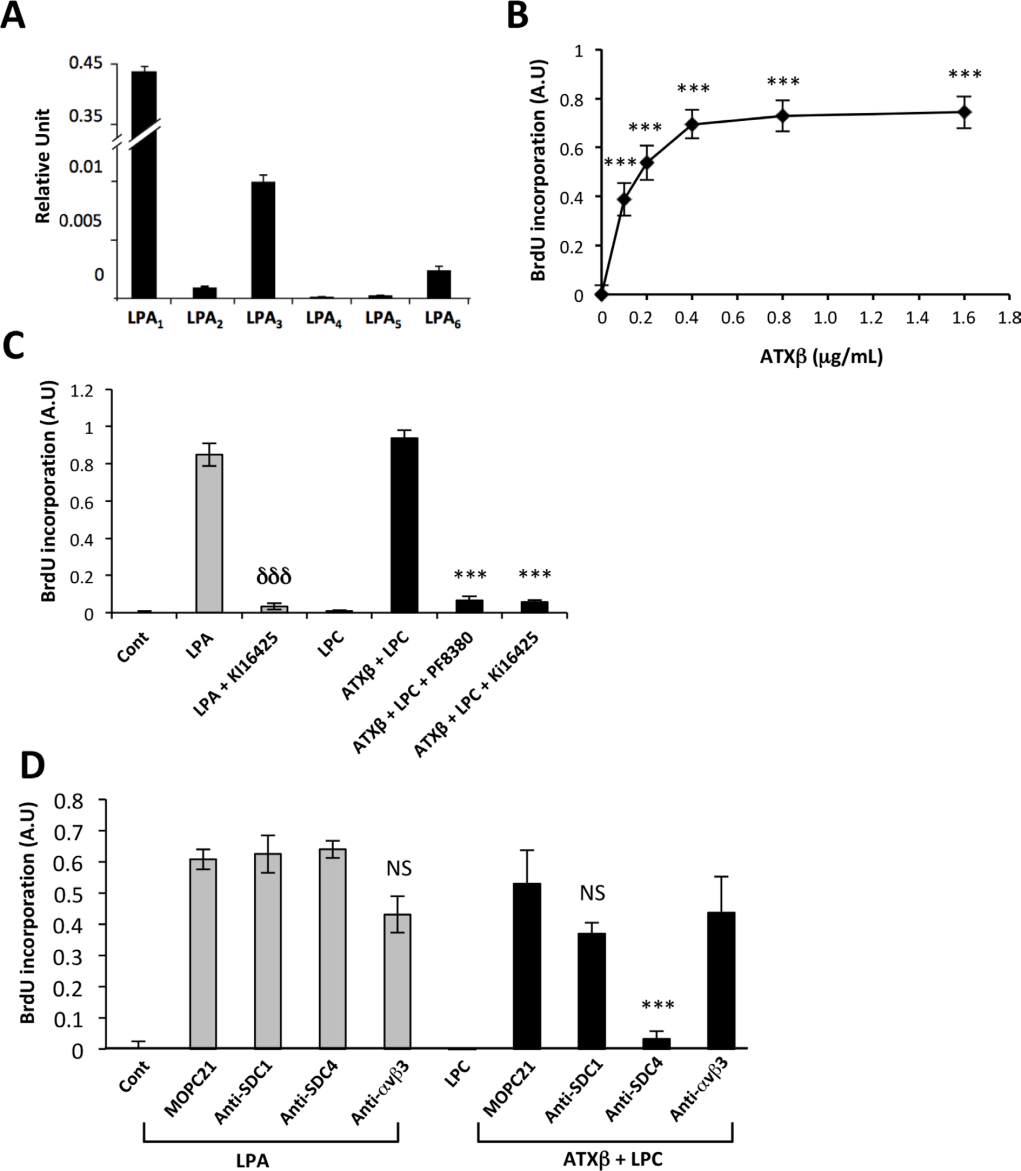
Blocking SDC4 inhibits ATXβ-induced cell proliferation (**A**) Real-time PCR analysis of lysophosphatidic acid receptor (LPA_1-6_) expression in MG63 cells. Values have been normalized to the housekeeping L32 gene. Data represent the mean ± SD of 2 independent experiments performed in triplicate. (**B**) Dose-response curve of MG63 cell proliferation generated in the presence of lysophosphatidylcholine (LPC; 1 µM) and increasing concentrations of exogenous ATXβ. Data represent the mean of BrdU incorporation ±SD of 3 independent experiments performed in triplicate (^***^P < 0.001 using 1-way ANOVA with a Bonferroni post-test). (**C**) Exogenous ATXβ induces MG63 cell proliferation through an LPA/LPA_1/3_-dependent mechanism. ATXβ (1 µg/mL), LPA (1 µM), Ki16425 (10 µM), LPC (1 µM), PF8380 (5 nM). Data represent the mean of BrdU incorporation ±SD of 3 independent experiments performed in triplicate (^***^: *P* < 0.001 vs LPA-treated cells and ^***^: *P* < 0.001 vs ATXβ + LPC -treated cells using 1-way ANOVA with a Bonferroni post-test). (**D**) Exogenous ATXβ induces MG63 cell proliferation through a SDC4-dependent mechanism. ATXβ (1 µg/mL), LPA (1 µM), LPC (1 µM) and indicated antibody (10 µg/mL). Data represent the mean of BrdU incorporation ±SD of 3 independent experiments performed in triplicate (^***^: *P* < 0.001 vs MOPC21- treated cells using 1-way ANOVA with a Bonferroni post-test).

LPA is a mitogenic factor on MG63 cells. MG63 cells express predominantly LPA_1_ followed by LPA_3_>> LPA_6_> LPA_2_ but do not express LPA_4_ and LPA_5_ (Figure 4A). This LPA receptor expression pattern explained why exogenous ATXβ+LPC induced MG63 cell proliferation in a dose-dependent manner (Figure 4B). As expected, LPA-induced cell proliferation was inhibited by Ki16425, a LPA_1/3_ antagonist (Figure 4C). In addition, exogenous ATXβ+LPC-induced cell proliferation was also inhibited by the both Ki16425 and PF8380, a blocker of lysoPLD activity of ATX; whereas LPC alone did not promote cell proliferation (Figure 4C). Remarkably, pretreatment of MG63 cells with anti-SDC4 antibodies significantly inhibited exogenous ATXβ+LPC-induced cell proliferation but not LPA-induced cell proliferation; whereas the isotypic antibody (MOPC21), and anti-SDC1 and anti-αvβ3 antibodies had no significant effect on either LPA-induced or soluble ATXβ+LPC-induced cell proliferation (Figure 5A–5D). Altogether, these results indicated that exogenous ATXβ induced MG63 cell proliferation through a LPA/LPA_1/3_–dependent signaling pathway mediated through an SDC4-dependent physical interaction of ATXβ with the cell surface.

### ATXβ controls breast cancer cell metastasis through an SDC4-dependent mechanism

**Figure 6:**
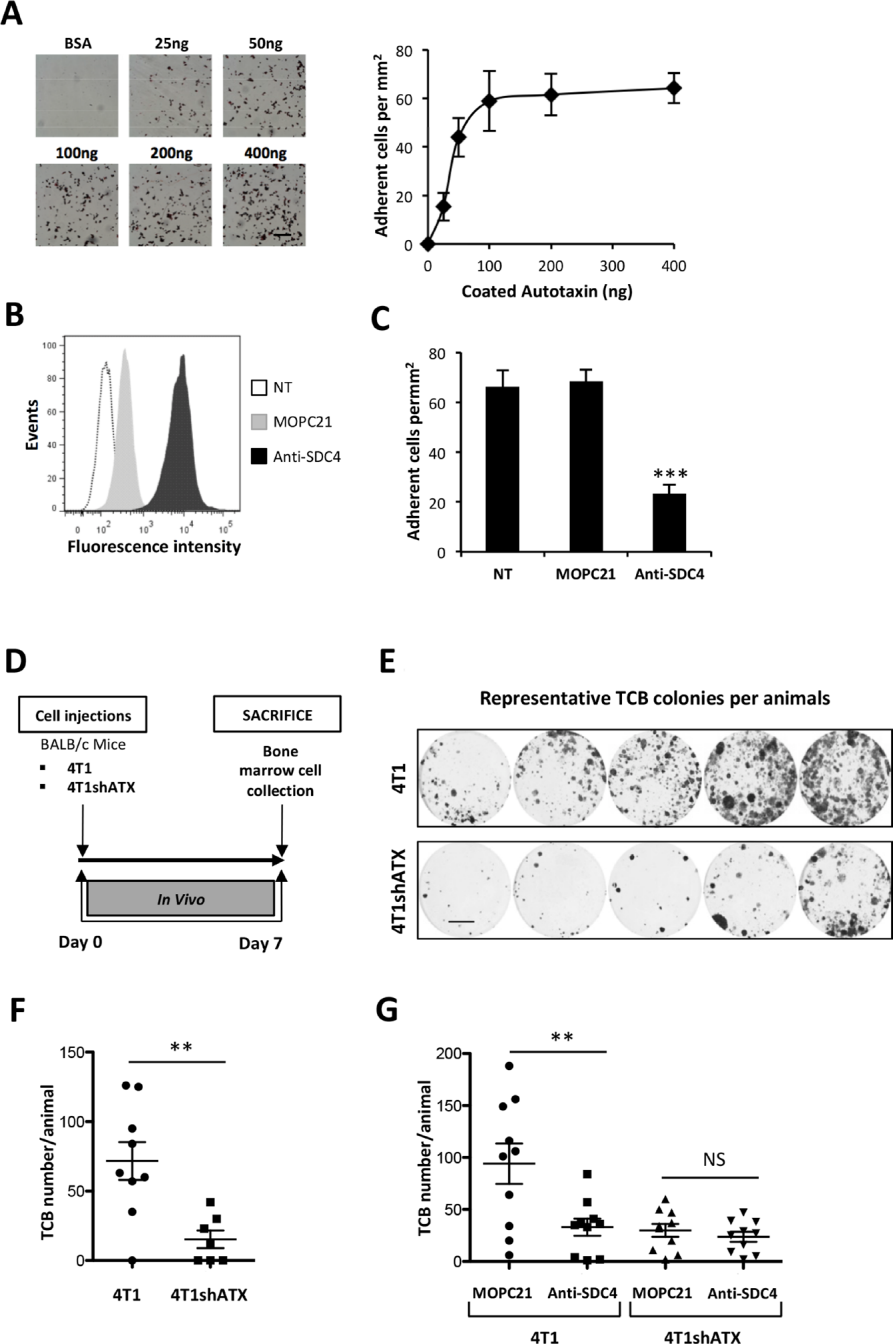
ATXβ controls breast cancer cell metastasis through an SDC4-dependent mechanism (**A**) 4T1 cell adhesion to increasing amounts of ATXβ, BSA (400 ng) was used as control (left panels). Data represent the mean of adherent cells/mm^2^ ±SD of adherent cells of 3 experiments performed in 8 replicates (right panel). (**B**) Flow cytometry detection of cell surface expression of syndecan-4 (SDC4) in 4T1 cells. Cells were immunostained with KY/8.2 monoclonal antibody (anti-SDC4) (black bar), or isotype control antibody MOPC21 (grey bar). NT: not treated cells (open bar). (**C**) Inhibition of 4T1 cell adhesion on ATXβ with KY/8.2 antibody (anti-SDC4). Indicated cell lines were preincubated for 1 h in the presence of KY/8.2 or MOPC21 antibodies (10 µg/mL). Data represent the mean of adherent cells/mm^2^ ±SD of adherent cells of 3 experiments performed in 8 replicates (^***^: *P* < 0.001, using 1-way ANOVA with a Bonferroni post-test). (**D**) Mice were injected with 4T1 or 4T1shATX cells. Bone marrow cells were collected at day 7 and cultured for 2 weeks in the presence of puromycin (1 µg/ml). (**E**) Colonies corresponding to tumor cells that colonized the bone (TCB) were fixed and stained with 20% methanol-crystal violet (v/v) (scale bar: 1 cm). (**F**) TCB colonies were counted. Results are expressed as mean of TCB ± SEM of *n =* 7 to *n =* 9 animals per group (^**^P < 0.01 using nonparametric Mann–Whitney test). (**G**) 4T1 and 4T1shATX cell lines were preincubated for 1 h in the presence of KY/8.2 (anti-SDC4) or MOPC21 antibodies (10 µg/mL) before injection into animals as described in D). TCB were obtained and quantified as described in (E) and (F). Data represent the mean of TCB ± SEM of *n =* 9 to *n =* 10 animals per group (^**^P < 0.01 using 1-way ANOVA with a Bonferroni post-test).

To evaluate whether SDC4 could control the ATX-dependent metastasis dissemination of cancer cells, we used the 4T1 breast cancer cells as they are communally used for their high metastatic potential to lungs and bone in syngenic BALB/C mice [30]. In addition we demonstrated previously that endogenous expression of ATX in these cells promotes metastasis formation to bone and lungs [31]. 4T1 cells adhered to ATXβ (Figure 6A) and expressed SDC4 (Figure 6B). Pretreatment of 4T1 cells with an anti-SDC4 antibody significantly inhibited their adhesion to ATXβ compared to cells pretreated with MOPC21 (Figure 6C). Remnant binding of 4T1 cells treated with anti-SDC4 antibody indicated that other adhesive molecules, among those αvβ3 integrin (Figure 1B), might contribute to ATXβ binding to the cell surface. Using an experimental setting allowing the quantification of early disseminated tumor cells to bone (TCB) [19, 32] (Figure 6D), we found that animals injected with 4T1shATX cells exhibited less TCB than animals injected with control 4T1 cells (Figure 6E–6F). This result was in total agreement with previously characterized role of tumoral ATX in 4T1 lung and bone metastasis formation [31]. Remarkably, animals injected with 4T1 cells previously incubated with anti-SDC4 antibody had a significantly lower number of TCB compared to mice injected with 4T1 cells treated with MOPC21 (Figure 6G). Additionally, animals injected with anti-SDC4-treated 4T1shATX cells did not show further increased inhibition in the number of TCB compared to mice injected with MOPC21-treated cells (Figure 6G). These results indicated that targeting SDC4 inhibited early steps on bone metastasis formation. Furthermore, our results determined that the prometastatic activity of SDC4 was dependent on ATX expression. The ATX/LPA axis is prevalent in 4T1 cells. Inhibition of ATX expression or pharmacological blockade of LPA signaling in these cells inhibited with equipotent potency the formation of metastasis both in soft and bone tissues [31, 33]. Therefore, our present study raised no evidence that our observations would be limited to the bone microenvironment but this remains subject of future studies. Nevertheless, our results determined for the first time that ATX and SDC4 are engaged in a reciprocal collaboration during early stage of 4T1 cell metastasis.

## DISCUSSION

In this study, we demonstrated that ATXβ interacts with the cell surface through a mechanism requiring the core protein of HS proteoglycan SDC4. Partnership between ATXβ and SDC4 demonstrated a remarkable impact *in vitro* since it is necessary for ATX-induced cancer cell proliferation, and *in vivo* since it controls breast cancer cell metastasis. ATX is a secreted protein known for its major contribution in LPA formation in the organism [34, 35]. ATX and LPA concentrations are substantially high in the blood circulation but surprisingly they are tightly buffered with no clear biological signification [36]. Increased systemic levels of ATX were found in a limited number of pathological situations, including in the blood circulation of patients with liver diseases (cancer, fibrosis, cirrhosis, viral infections) or in the synovial liquid of rheumatoid arthritis patients [14, 37]. Also in these cases, the pathophysiological impact of increased ATX concentration is totally unknown. In contrast, interactions of ATX with cell surface adhesive molecules may be critical for controlling diverse pathophysiological processes. ATX interacts with α4β1 and αvβ3 integrins, mediating CD4 T-lymphocyte migration into secondary lymphoid organs and cancer cell metastasis, respectively [18, 19, 22].

Distinct expression signatures of LPA receptors and LPA-producing enzymes was shown to distinguish large cancer entities from one another [38]. However, the direct link between LPA signaling and patient disease- or recurrence-free survival that would lead to new anti-LPA receptor therapies has not been firmly characterized yet. Expression of various integrins and HS chains in cancer entities increases the complexity of LPA signaling as they potentially contribute to ATX specific docking sites controlling the bioavailability of LPA to its receptors. Furthermore, LPA-degrading enzymes such as lipid phosphate phosphohydrolases (LPPs) may also contribute to LPA signal duration and strength as observed in ovarian cancer [39]. Our cell line study focused on three highly metastatic cancer entities, osteosarcoma, breast and prostate cancers that were shown to express endogenous ATX [38]. Extending our observations to other cancer entities would require further investigation especially on the category of cancers expressing high ATX levels such as melanoma, kidney carcinoma, non small cell lung carcinoma, pancreatic carcinoma and neuroblastoma [38].

All five isoforms of ATX (α, β, γ, δ, ε) possess the somatomedin B-like 2 domain containing amino acids E^109^ and H^117^ involved in the binding to integrins [17, 37]. As specificity, ATXα possesses an insertion sequence of 52 polybasic amino acids, also found in ATXε, which explains the binding of ATXα to HS chains [21]. Recently, binding of ATXβ to β3 integrin family members (αIIbβ3, αvβ3) was discovered [17]. Activated platelets but not resting platelets bind to ATXβ, indicating the necessity of conformational activation changes of αIIbβ3 integrin in this process [17]. ATX taken up by platelets from the blood circulation is stored in α-granules and secreted following tumor cell-induced platelet aggregation supporting metastasis even of ATX-null breast cancer cells [19]. ATXβ also binds to α4β1 integrin through a putative domain containing the leucine-aspartic acid-valine amino acid sequence since it is recognized as an α4β1 binding motif [22]. Here, we found that the HS proteoglycan SDC4 was required for binding of ATXβ to αvβ3 integrin low expressing cancer cells. ATX isoforms share more than 93% of identity in their amino acid sequence. However, each isoform has a unique feature even modest such as a four amino acid deletion in exon 19 between ATXβ and ATXδ [37] that could be sufficient for driving specificity including to cell binding. Because we used only ATXβ as a source of recombinant proteins our observations could not be generalized to all forms of ATX.

SDC4 core protein is associated with both HS and chondroitin sulfate chains [40]. Treatment of cancer cells with chondroitinase ABC had no impact on ATXβ cell binding. In contrast, heparinase (I, II or III) treatments increased cell binding, indicating that instead of promoting ATXβ binding as observed for ATXα, HS chains partially restricted ATXβ cell interaction. These results supported the existence of different mechanisms involved in the binding of growth factors (midkine, pleiotrophin and basic fibroblast growth factors) to SDC4 on mammary epithelial cells that are mediated through cooperation between HS and chondroitin sulfate chains [41]. Our observation suggests that increased HS chain degradations might enhance to ATX binding to the cell surface, contributing to pathophysiological processes. Enzymatic activity of heparanase degrading specifically HS chains of HSPG is involved in tumor progression and metastasis [42]. Increased expression of heparanase in the mammary epithelium of MMTV-driven gene transgenic mice promotes the growth of cancer cells and lung metastasis formation, supporting the notion that increased matrix degradation by heparanase contributes to cancer spreading [43]. Heparanase treatment also mediates clustering of SDC, including SDC4, increasing cell adhesion and spreading through intracellular recruitment of PKCα, Rac1 and Src [44]. The characterization of potential links between LPA pro-metastatic action and HSPG clustering would deserve special investigations.

The recent discovery of ATX binding to cell adhesion molecules brings to a new function for ATX as an LPA docking protein. In the organism, LPA is essentially linked to albumin and gelsolin, which provide physical protection against phospholipase action and conformational presentation to LPA receptors [45]. Binding of ATX to the cell surface potentially makes LPA immediately available to its receptors and fast activation of downstream intracellular signaling pathways. In addition, Fulkerson and colleagues showed that lysoPLD activity generated by exogenous ATXβ was increased in conditioned media of CHO cells expressing functionally active αIIbβ3 integrin compared to parental cells [17]. Furthermore, the use of 7E3, a β3 integrin-blocking antibody, almost totally inhibited lysoPLD activity from exogenous ATXβ in culture media of αIIbβ3 integrin-expressing cells [17]. These results support a novel provocative hypothesis since ATX becomes active once it is properly located at the cell surface. Houben and colleagues also found an increase in lysoPLD activity when soluble ATXα was incubated with heparin [21]. ATX was recently found associated with apolypoprotein A and apolypoprotein B in lipoprotein(a) (Lp(a)) in the plasma of patients with calcific aortic valve stenosis with increased lysoPLD activity in the Lp(a) fraction in the circulation [46, 47]. Here, we found that silencing of SDC4 expression totally inhibited MG63 cell proliferation induced by exogenous ATXβ. Altogether, these results strongly support the hypothesis that physical interaction of ATXβ with adhesive molecules makes functional changes in ATX required for LPA biological functions.

During this study we found no evidence for a direct interaction between SDC4 and ATXβ. Nevertheless, our new data revealed that the VS52 sequence of SDC4 extracellular domain is functionally important for cell binding to ATXβ that is mediated through an undetermined partner. Our results also suggested the existence of a cryptic ATX-binding motif, unveiled upon heparinases treatment, likely situated on a SDC4 co-receptor. Indeed, studies of McFall and Rapraeger demonstrated that a site of the extracellular domain of SDC4 core protein, located in the VS52 sequence, interacts with another cell surface receptor that is still not yet characterized [28, 48]. Differential expression of this undefined partner, integrin or other, as well as specific modifications of HS chains may contribute to differential binding capacity of cells even expressing high levels of SDC4. SDC4 connects with αvβ3 and α5β1 integrins controlling integrin recycling and cell adhesion through highly dynamic mechanisms that involve Src phosphorylation of SDC4 intracellular domain [49]. Cooperation between integrins and exogenous ATXβ was recently shown to drive directional cell migration, where binding of ATXβ to integrin enabled the uptake and subsequent redistribution of ATXβ to the leading edge of migrating cells [18]. It is reasonable to hypothesize that SDC4 may functionally contribute to this process.

In conclusion, our results determined that ATX and SDC4 are engaged in a reciprocal collaboration in cancer cell metastasis. Furthermore, our study provides a rationale for the development of novel anti-ATX therapies that besides small molecules that block lysoPLD activity new strategies should also target ATX cell surface binding motifs.

## MATERIALS AND METHODS

### Cell culture and reagent

Human breast cancer cell lines MDA-MB-231, T47D, MCF-7 and ZR-75-1, and mouse B16-F10 melanoma cells were obtained and cultured as recommended by American Type Culture Collection (ATCC; Gaithersburg, MD, USA). Characterization of MDA-B02 human breast cancer cells and culture conditions were described previously [50]. CHO-β3wt and CHO-Dhfr cells were generated and cultured as described previously [25]. Both human and murine cell lines were obtained from and cultured as recommended by American Type Culture Collection (Gaithersburg, MD). Briefly, human prostate DU145 and PC3 cancer cells were culture in Dubelcco’s Modified Eagle Medium (DMEM) (Gibco, Thermofisher, Dardilly, France) and Ham’s F12-K Medium (Gibco, Thermofisher, Dardilly, France), respectively. Human KHOS and MG63 osteosarcoma cells were cultured in Eagle’s Minimum Essential Medium (Gibco, Thermofisher, Dardilly, France) and DMEM, respectively. Mouse mammary 4T1 carcinoma cells were cultured in RPMI-1640 (Gibco, Thermofisher, Dardilly, France). 4T1shATX cells were generated and cultured as described previously [31]. LM609 monoclonal antibody directed against human integrin αvβ3 was from Merck Millipore (Darmstadt, Germany). Mouse monoclonal antibodies directed against human syndecan-1 (SDC1; DL-101 clone) and human SDC4 (5G9 clone) were from Santa Cruz Biotechnology (Santa Cruz, CA, USA). Rat monoclonal anti-mouse syndecan-4 (KY/8.2) was from Becton Dickinson Biosciences (Franklin Lakes, NJ, USA) and MOPC21 antibody was from ICN Pharmaceuticals (Paris, France). FITC fluorochrome-conjugated anti-mouse IgG and anti-rat IgG antibodies were from Invitrogen (Carlsbad, CA, USA). 1-lysophosphatidylcholine (1-Lyso-PC 18:1), lysophospahtidic acid (LPA, Oleoyl C18:1) and Ki16425 were from Interchim (Montluçon, France). PF-8380 (ATX inhibitor III) was from Merck Millipore (Darmstadt, Germany) and Cayman Chemical (Interchim, Montluçon, France). Recombinant active ATXβ was generated as a double myc- and His-tag protein and purified as described previously [19]. Synthetic peptides were purchased from Proteogenix (Schiltigheim, France).

### Transient transfection

MG63 cells were transiently transfected with 25 nM of silencer select predesigned SiRNA against SDC1, SDC4, GPC1 and GPC4 were obtained from Life Technologies (ThermoFisher, Dardilly, France), 25 nM of BLOCK-iT Alexa fluor-555 was used as negative control using Lipofectamine RNAiMAX Reagent (ThermoFisher, Dardilly, France) following the manufacturer’s protocol. Cells were used for *in vitro* experiments 48 h post-transfection.

### Flow cytometry analysis

Cell surface expression of αvβ3 integrin and SDC4 was analyzed via FACscanCantoII (BD Biosciences, Franklin Lakes, NJ, USA). Briefly, cells were detached using accutase (Sigma Aldrich, Saint Louis, USA), washed in a PBS1X-BSA 0.3% solution and kept on ice for the remaining procedure. Cells (5 × 10^5^ cells/ml) were then incubated 1 h at 4° C with primary anti-αvβ3 or anti-SDC4 antibodies (10 µg/ml), washed with PBS1X-BSA0.3%, and incubated with FITC flurochrome-conjugated anti-mouse IgG or anti-rat IgG (1:2000 dilution). Cells were finally washed and suspended in PBS1X-BSA0.3% for analysis.

### Cell adhesion assay

Cell adhesion assays were carried out as previously described [25]. Plates were coated with bovine serum albumin or recombinant ATXβ. Cells were detached and resuspended in HEPES-buffered Tyrode solution supplemented with 2 mM Mn2+ (10^5^ cells in 100 μL of buffer), rested for 1 h at 37° C, and seeded into coated plates for 1 h. Attached cells were fixed, stained with a solution of crystal violet, and counted under the microscope. Results were expressed as the number of attached cells per mm^2^.

### Surface plasmon resonance spectroscopy

Experiments were performed on a Biacore T200 (GE Healthcare, Uppsala, Sweeden). Sensor Chip CM5 was conditioned following the manufacturer’s notice instruction 22-0607-37AF (GE Healthcare, Uppsala, Sweeden). Briefly, capture of ATXβ was performed on the Fc2 compartment at a concentration of 10 µg/mL (flow rate 5 µL/min for 360 s). The same procedure was applied to Fc1 compartment but in absence of recombinant proteins. Both compartments were washed with the running buffer (TRIS pH 8, NaCl 150 mM, surfactant P20 0.05% v/v) at a flow rate of 5 µL/min for 360 s. Synthetic peptides were used as analytes at concentrations of 1µM, 10 µM and 50 µM, at a flow rate of 30 µL/min for 120 s. Analyte dissociations were measured during 200 s.

### Cell proliferation assay

Experiments were carried out in conditions described previously [10]. Briefly, MG-63 cells (8 × 10^3^) were seeded into 48-well plates and cultured in complete medium for 12 h and synchronized in the presence of serum-free medium for 24 h. Cells were then treated with LPA (1 µM) or ATXβ (at indicated concentration) + LPC (1 µM) in the presence or absence of specific inhibitors or antibodies (10 µg/mL) for 24 h. Cells were synchronized in the presence of serum-free medium for 24 h. Then cell proliferation was evaluated following BrdU incorporation for 7 h and the use of a cell proliferation ELISA kit (Roche Diagnostics, Meylan, France).

### Reverse transcription-polymerase chain reaction (RT-PCR)

Total RNA was extracted using Nucleospin RNAII kit (Macherey-Nagel) and cDNA was synthesized using iScript cDNA Synthesis kit (Biorad), as previously described [33]. Expression of LPA receptors, syndecans (SDC) and glypicans (GPC) was quantified by real-time quantitative RT-PCR in an Eppendorf MastercyclerH RealPlex (Invitrogen) using the SYBRH Green PCR kit (Life Technologies). Quantifications were normalized to corresponding L32 RNA. The cDNAs were amplified by PCR for 35 cycles with specific PCR primers listed in Table 2. Each cycle consisted of 10 s of denaturation at 95° C, 15 s of annealing at 67° C, and 10 s of extension at 72° C.

**Table 2:** List of primers

Gene	Fw/Rv	Sequence 5′-3′	Location	Fragment size	T° C	SLOPE	INT.
***LPAR1***	Fw	AATCGAGAGGCACATTACGG	643−662	181	58	−3.596	15.73
	Rv	CTGTAGAGGGGTGCCATGTT	822−803				
***LPAR2***	Fw	CGCTCAGCCTGGTCAAGACT	954−933	108	60	−3.871	A
	Rv	TTGCAGGACTCACAGCCTAAAC	846−865				
***<LPAR3***	Fw	TGCTCATTTTGCTTGTCTGG	479−498	165	60	−3.987	19.14
	Rv	ATGATGAGGAAGGCCATGAG	644−625				
***LPAR4***	Fw	GCCTGCTACTCTGTCTCAAATTGG	1234−1258	200	60	−3.322	23.64
	Rv	GCAAGGCACAAGGTGATTGG	1434−1415				
***LPAR5***	Fw	CTCGGTGGTGAGCGTGTACATG	433−464	211	60	−4.378	22.96
	Rv	GCGTAGCGGTCCACGTTGAT	644−624				
***LPAR6***	Fw	AAATTGGACGTGCCTTTACG	1190−1171	115	60	−3.972	19.73
	Rv	TAACCCAAGCACAAACACCA	1075−1054				
***SDC1***	Fw	CCAGCAGATGAGCATGGTC	2882−2863	179	60	−3.781	21.33
	Rv	ACGTTTCAGCGACTCCG	2703−2719				
***SDC2***	Fw	TCATTAACACCAGTCTGCAACA	2173−2152	137	67	−3.710	22.16
	Rv	TGCAATTTGAAGGTACGAGTAG	2057−2036				
***SDC3***	Fw	GCCATGAACTCAGGACAGGT	4888−4907	127	60	−3.981	26.4
	Rv	CAGTGTCCCTCCCTCCTGT	4779−4761				
***SDC4***	Fw	CCTCAGTTGCACTAACCACG	2095−2114	139	60	−3.679	22.85
	Rv	AGCTGAGGCTGTGACTCGTT	2234−2215				
***GPC1***	Fw	AGGCCAAGGACTGACTTTGC	1922−1941	229	60	−4.142	26.39
	Rv	GAGGTCCCTGAAATACATGGC	2131−2151				
***GPC2***	Fw	CACCTGCTGTTCCAGTGAGA	198−217	179	58	−3.638	24
	Rv	GAGCTGGGTCAGAGAGTGCT	376−357				
***GPC3***	Fw	CCTGATTCAGCCTTGGACAT	500−519	240	58	−3.911	22.14
	Rv	TCCCTGGCAGTAAGAGCAGT	665−646				
***GPC4***	Fw	CAGGGTCTGGGAGCCAAGT	304−322	128	60	−3.672	23.79
	Rv	GCACAGTGCTGGACATTGACA	432−412				
***GPC5***	Fw	GGGCTGCCGGATTCG	153−167	73	60	−4.182	21.73
	Rv	CTGGTGCAACATGTAGGCTTTT	205−226				
***GPC6***	Fw	ATCGGGGCTGTGATTCTTC	628−646	175	60	−4.331	24.15
	Rv	TGAATCCCTTGGCACCGTA	471−489				
***L32H***	Fw	CAAGGAGCTGGAAGTGCTGC	340−359	100	67	−3.546	15.43
	Rv	CAGCTCTTTCCACGATGGC	440−422				

Fw/Rv: Forward/Reverse; Localization; T° C: hybridation temperature; INT.: Interception.

### Early bone colonization in breast cancer cell experiments

All protocols involving animal experimentations were approved by The Institutional Animal Care and Use Committees CEEA-55 in Lyon, France (authorization number: DR2016-20v2) and the French Ministère de l’Education Nationale de l’Enseignement Supérieur et de la Recherche (authorization number: APAFIS#5136-2016042115384192v2; Validation date: 10/2016). Animals were purchased from Janvier Labs (Le Genest-Saint-Isle, France) and housed under closed barrier conditions as previously described [31]. Briefly, 4T1 or 4T1shATX cells (5 × 10^5^ in 100 µL of phosphate-buffered saline) were injected in the tail artery of 6-week-old BALB/C mice as previously described [31]. Seven days post-injection, the mice were euthanized by cervical dislocation and the hindlimbs were dissected. Bones were chopped and treated with 0.25 mg/mL collagenase for 2 h at 37° C. The cell suspension was washed with phosphate-buffered saline and resuspended in complete media supplemented with 6-thioguanine (10 µg/ml). After 2 weeks, clones were fixed and stained with a solution of crystal violet and counted.

### Statistical analysis

Differences between groups were determined by 1-way or 2-way analysis of variance (ANOVA), followed by a Bonferroni post-test using GraphPad Prism v5.0c software. Single comparisons were carried out using the nonparametric Mann–Whitney *U* test. *P* < 0.05 was considered statistically significant.

## SUPPLEMENTARY MATERIALS FIGURES



## Abbreviations

ATX: Autotaxin
CS-HSPG: Cell surface heparan sulfate proteoglycan
DMEM: Dubelcco’s Modified Eagle Medium
E^109^: Glutamic acid amino acid number 109
H^117^: Histidine amino acid number 117
HS: Heparan sulfate
HSPG: Heparan sulfate proteoglycan
GPC: Glypican
GPC1: Glypican-1
GPC4: Glypican-4
Lp(a): Lipoprotein (a)
LPA: Lysophosphatidic acid
LPA_1-6_: Lysophosphatidic acid receptor 1 to 6
LPC: Lysophospatidylcholine
LysoPLD: Lysophospholipase D
RT-PCR: Reverse transcription-polymerase chain reaction
SDC: Syndecan
SDC1: Syndecan-1
SDC4: Syndecan-4
shATX: Anti-ATX small hairpin siRNA
TCB: Tumor cells disseminated to bone
