# Supplementary material for: Autotaxin-β interaction with the cell surface via syndecan-4 impacts on cancer cell proliferation and metastasis
Source: Oncotarget. 2018 Sep 4;9(69):33170–85. doi: 10.18632/oncotarget.26039 (PMC6145688; doi:10.18632/oncotarget.26039)
Supplement: Supplementary file 1 [file oncotarget-09-33170-s001.pdf]

Autotaxin-β interaction with the cell surface via syndecan-4 impacts on cancer cell proliferation and metastasis

SUPPLEMENTARY MATERIALS

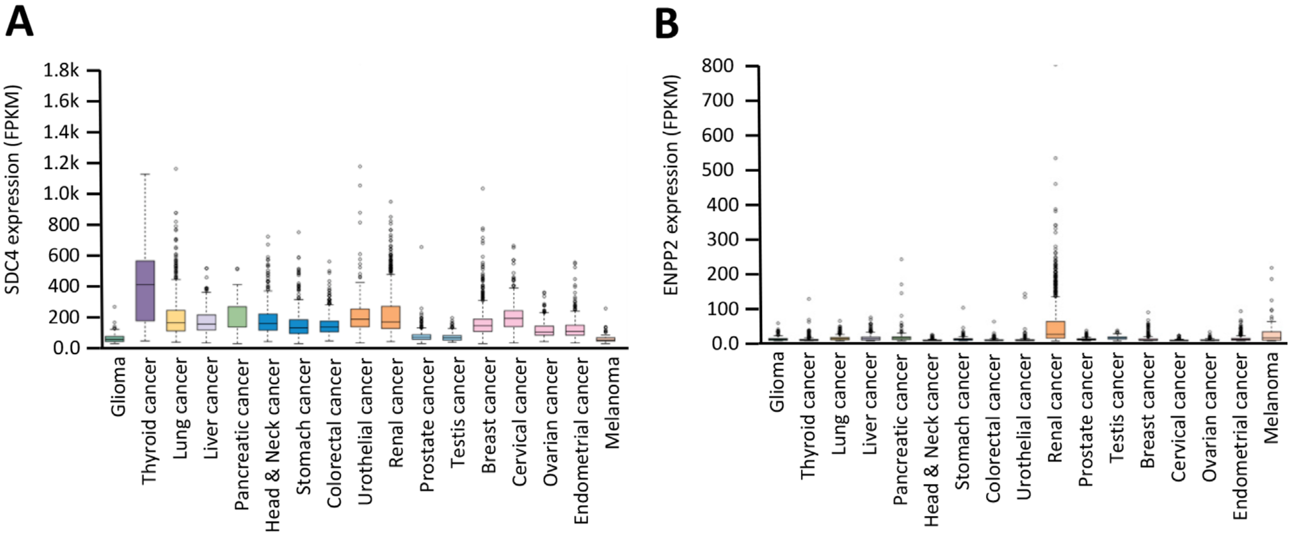

Supplementary Figure 1: Expression of SDC4 and ENPP2 mRNAs in human primary tumors. Data were obtained from the pathology atlas database (https://www.proteinatlas.org). Extracting mRNA data from the TCGA database using entries: (**A**) ENSG00000124145 and (**B**)ENSG00000136960.

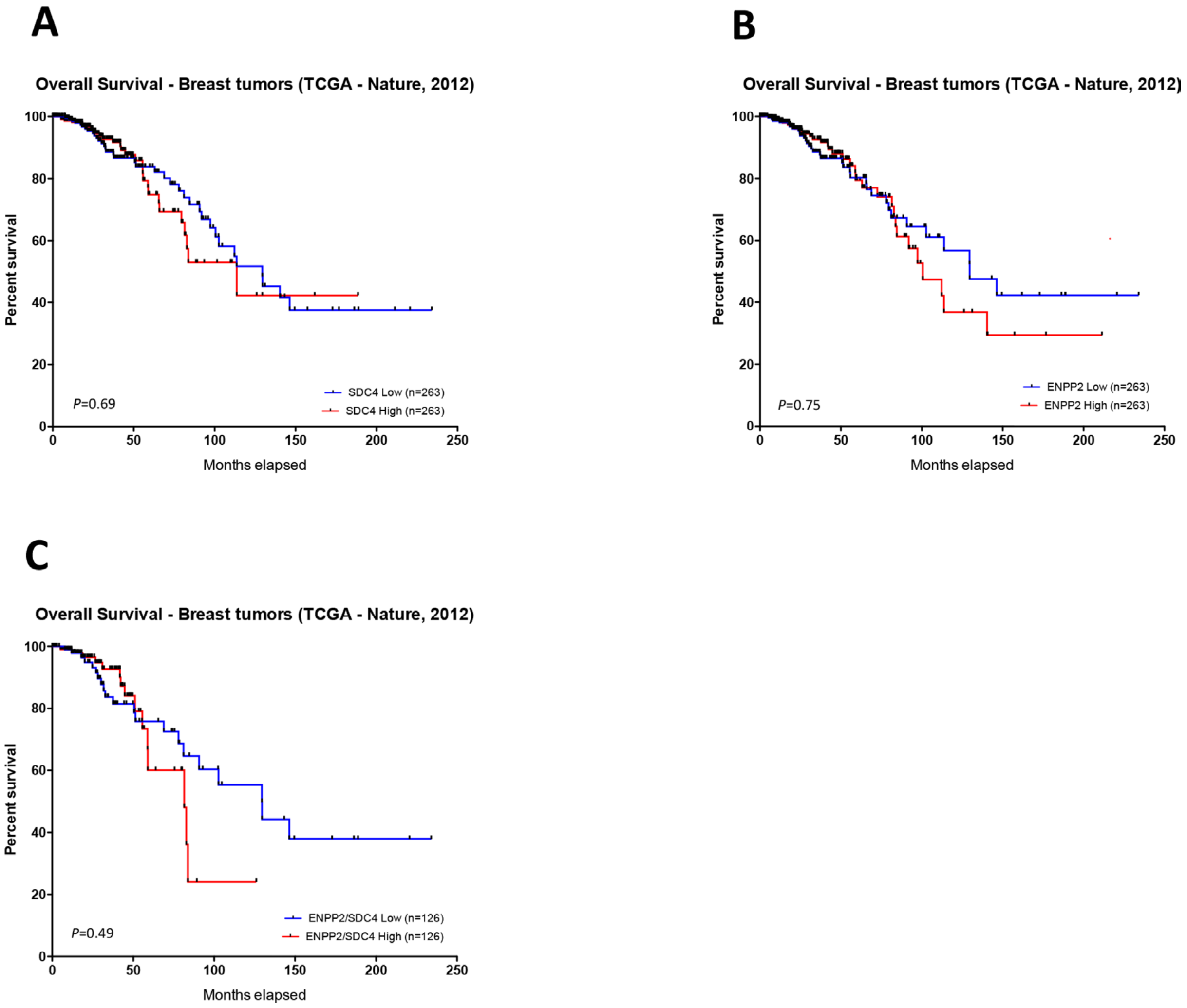

Supplementary Figure 2: Kaplan–Meier analyses of (**A**) SDC4, (**B**) ENPP2 and (**C**) combined ENPP2/SDC4 expression on breast tumor-bearing patient overall survival. Data from the TCGA dataset [27] were extracted using cbioportal. Sampling was based on the median value of each gene.
